# Calendar

**DOI:** 10.1155/S1463924689000052

**Published:** 1989


					S. Schonfield et al. Evaluation of the Nova Stat Profile
4. TOFFALETTI,J., BLOSSER, N., KIRVAN, K. Effects ofStorage,
Temperature and Time before Centrifugation on Ionized
Calcium in Blood Collected in Plain Vacutainer Tubes and
Silicone-Separator (SST) Tubes. Clin. Chem 1984; 30/4."
553-556.
5. DEMING, W. E. Statistical Adjustment ofData. John Wiley &
Sons, New York, N.Y. 1943: p. 184.
6. SMITH, S. C. H. International ionised calcium meeting-
report. News Sheet Assoc. Clin. Biochem., 1986; 278:10-12.
7. JOHNSON, M. T., SAVORY, J., WILLS, M. R. Evaluation ot"
the Nova Stat Profile 1. Am. Clin. Prod. Rev., 1986; Oct.
8. BOWERS, G. N. JR, BRASSARD, C., SENA, S. F. Measurement
ofionised calcium in serum with ion-selective electrodes; a
mature technology that can meet the daily service needs.
Clin. Chem. 1986; 32: 1437-1447.
9. BURNS, J., LAW, R. W. G., PATERSON, C. R. Measurement
of plasma ionised calcium: comparison of calcium-titrated
sodium heparin and lithium heparin as anticoagulants..
Med. Lab. Sci. 1986; 44: 187-189.
10. Butler, S.J., Payne, R. B., Gunn, I. R., et al. Correlation
between serum ionised calcium and albumin concentration
in two hospital populations. Br. Med. j. 1984; 289: 248-50.
Calendar
FEBRUARY
8-9 February 1989 Flow Analysis Methods: London, UK.
Contact Analytical Division, RSC, Burlington House,
Piccadilly, London W1V 0BN.
MARCH
6-10 March 1989 Pittsburgh Conference 1989: Atlanta, GA,
USA. Contact Pittsburgh Conference, Suite 322, 12
Federal Drive, Pittsburgh, PA 15235, USA.
7-9 March 1989 Workshop: StatisticsforAnalytica.l Chemists:
Eastbourne, UK. Contact Christine Robinson, Statistics for
Industry (UK) Ltd, 4 Victoria Avenue, Knaresborough,
North Yorks HGF 9EU, UK.
21-22 March 1989 Research and Development Topics in
Analytical Chemistry: Dublin, Ireland. Contact Analytical
Division, Royal Society ofChemistry, Burlington House,
Piccadilly, London W1V 0BN.
APRIL
4-7 April 1989 RSC Annual Congress: Hull, UK. Contact
Gina B. Howlett, Conference Officer, RSC, Burlington
House, Piccadilly, London N1V 0BN. The Analytical
Division session will be on Process Analysis and Information
Management.
11-14 April 1989 VIIth International Symposium on Electro-
analysis and Sensors in Biomedical, Environmental and Industrial
Sciences: Loughborough, UK. Contact Dr A. G. Fogg,
Chemistry Department, University of Loughborough,
Leicestershire LE11 3TU.
JUNE
25-30 June 1989 Eurosensors III and 5th International
Conference on Sensors and Actuators: Montreux, Switzerland.
Contact Eurosensors (Transducers '89), COMST S.A.,
PO Box 415, 1001 Lausanne 1, Switzerland.
JULY
30 July-5 August 1989 SAC 89." Cambridge, UK. Contact
Analytical Division, Royal Society ofChemistry, Burling-
ton House, Piccadilly, London W1V 0BN.
SEPTEMBER
11-13 September 1989 RSC/SCI/IOP Joint International
Symposium on Surface Analysis Techniques and Applications:
Manchester, UK. Contact Mrs E. S. Wellingham, Field
End House, Bude Close, Nailsea, Bristol BS19 2FQ.
25-27 September 1989 Sensors and their Applications IV:
Canterbury, UK. The Meetings Officer, Institute of
Physics, 47 Belgrave Square, London SWlX 8QX.
25-28 September 1989 Third International Symposium on
Kinetics in Analytical Chemistry: Dubrovnik, Yugoslavia. Con-
tact Professor Gordana A. Milonovi. Department of
Chemistry, University of Belgrade, KAC PO Box 550,
11001 Belgrade, Yugoslavia.
26-28 September 1989 The British Laboratory Week:
Olympia, London. Including Laboratory 89, Computer
Aided Sciences, Bio 89, Medical Laboratory Sciences and
Analyticon. Contact Curtis Steadman and Partners, The
Hub, Emson Close, Saffron Walden, Essex CB10 1HL.
27

				

